# Client and provider factors associated with integration of family planning services among maternal and reproductive health clients in Kigoma Region, Tanzania: a cross-sectional study, April–July 2016

**DOI:** 10.1186/s12978-018-0593-5

**Published:** 2018-09-12

**Authors:** M. M. Dynes, E. Bernstein, D. Morof, L. Kelly, A. Ruiz, W. Mongo, P. Chaote, R. N. Bujari, F. Serbanescu

**Affiliations:** 1 Centers for Disease Control and Prevention, Division of Reproductive Health, Atlanta, USA; 2 Centers for Disease Control and Prevention, Division of Reproductive Health (CDCF Contractor), Atlanta, USA; 3 EngenderHealth, Washington, DC, USA; 4 Regional Medical Officer, Kigoma, Kigoma Region Tanzania; 5 AMCA Inter Consult, Dar es Salaam, Tanzania

**Keywords:** Family planning, Family planning counseling, Family planning integration, Contraception, Contraceptive methods, Sub-Saharan Africa, Tanzania, Multilevel modeling

## Abstract

**Background:**

Integration of family planning (FP) services into non-FP care visits is an essential strategy for reducing maternal and neonatal mortality through reduction of short birth intervals and unplanned pregnancies.

**Methods:**

Cross-sectional surveys were conducted across 61 facilities in Kigoma Region, Tanzania, April–July 2016. Multilevel, mixed effects logistic regression analyses were conducted on matched data from providers (*n* = 330) and clients seeking delivery (*n* = 935), well-baby (*n* = 272), pregnancy loss (PL; *n* = 229), and other routine (postnatal, HIV/STI, other; *n* = 69) services. Outcomes of interest included receipt of FP information and a modern FP method (significance level *p* < 0.05).

**Results:**

Clients had significantly greater odds of receiving FP information if the primary reason for seeking care was for PL versus (vs) any other types of care (aOR 1.97), had four or more pregnancies vs fewer (aOR 1.78), and had had a FP discussion with their partner vs no FP discussion (aOR 1.73). Clients had lower odds of receiving FP information if they were aged 40–49 vs 15–19 (aOR 0.50) and reported attending religious services at least weekly vs less frequently (aOR 0.61). Clients of providers who perceived that in-service training had helped vs had not helped job performance (aOR 2.27), and clients of providers having high vs low recent FP training index scores (aOR 1.58) had greater odds of receiving FP information.

Clients had greater odds of receiving a modern method when they received information on two or more vs fewer methods (aOR 7.13), had had a FP discussion with their partner vs no discussion (aOR 5.87), if the primary reason for seeking care was for PL vs any other types of care (aOR 4.08), had zero vs one or more live births (aOR 3.92), made their own FP decisions vs not made own FP decisions (aOR 3.17), received FP information from two or more vs fewer sources (aOR 3.12), and were in the middle or high vs the low wealth tercile (aOR 1.99 and 2.30, respectively). Well-baby care clients, Other routine services clients, and married clients had significantly lower odds of receiving a method (aOR 0.14; aOR 0.08; and aOR 0.41, respectively) compared to their counterparts.

**Conclusions:**

Strategies that better integrate FP into routine care visits, encourage women to have FP discussions with their partners and providers, increase FP training among providers, and expand FP options and sources of information may help reduce the unmet need for FP, and ultimately lower maternal and neonatal mortality.

## Plain English summary

Integrating family planning (FP) services into non-FP care visits is an important approach to reduce maternal and newborn deaths through reduction of short birth intervals and unplanned pregnancies. Analyses of matched interviews with 1505 clients and 330 providers in Kigoma Region, Tanzania identified key factors for clients receiving FP information and a modern method. Clients were more likely to receive FP information if they were at the facility for pregnancy loss, had four or more pregnancies, and had discussed FP with their partner. Clients were less likely to receive FP information if they were 40 to 49 years old and attended religious services at least weekly. Providers were more likely to provide FP information if they perceived in-service training had helped their job performance, and had received more recent FP training. Clients were more likely to receive a FP method if they received information on two or more FP methods, had discussed FP with their partner, were at the facility for pregnancy loss, had no live births, made their own FP decisions, received FP information from two or more sources, and were in the middle or high wealth groups. Well-baby care clients, Other services clients, and married clients were less likely to receive a FP method compared to others. FP services need to be better integrated into well-baby and other routine outpatient services. Interventions are needed that encourage women to have FP conversations with their partners and providers, expand FP options and sources of information, and encourage providers to ask about reproductive health goals at each client encounter.

## Introduction

Sub-Saharan Africa experiences disproportionately high maternal mortality in comparison to other regions, contributing to two-thirds of the world’s total maternal deaths in 2015 [1]. Tanzania had an estimated 8200 maternal deaths in 2015, the fourth highest number of maternal deaths in sub-Saharan Africa and the sixth highest number in the world [1]. Family planning (FP) can reduce the risk of maternal and neonatal mortality by reducing the number of short birth intervals, as well as unplanned and high-risk pregnancies [2–7]. In 2016, the total fertility rate (TFR) in Tanzania’s Western Zone, which includes Kigoma Region, was 6.7 births per woman – the highest TFR in all of Tanzania [8].

Research in Western and African clinical settings has found that quality FP counseling—identified as six distinct elements by the Bruce Framework [9]—has a significant impact on FP knowledge and use [10, 11]. To increase postpartum FP uptake and reduce unmet need, FP counseling and provision are routinely recommended for integration into antenatal, labor and delivery, and postnatal care services [12–17]. However, evidence varies on whether the increase in FP use relates to the type of care services in which FP services are being integrated [13, 23, 27], number of times counseling is provided [17–20], whether the FP counseling is provided during the antenatal versus postpartum period [15, 17–21], and whether modern contraceptive supplies are available at the time/onsite. Moreover, some studies have found positive effects while others have found no effect [16, 21, 22].

Furthermore, many factors may influence FP uptake even when services are integrated. Necessary infrastructure improvements, increased staff resources, and availability of FP supplies rarely accompany the integration of FP services into other health services [14, 19]. Long wait times, unclear pathways to FP providers, and lack of privacy deter women from accepting a FP referral [23]. In health facility assessments in Kigoma Region, Tanzania in 2015, only 57% of dispensaries had implants and only 39% had intrauterine devices (IUD) available to clients [24].

### Client predictors of FP use

Women’s individual, sociodemographic characteristics, and life circumstances also influence FP uptake. Partner communication and support, self-efficacy, intensity of antenatal care visits, and previous FP use have been found to be positively associated with FP uptake [16, 20, 25]. Lower wealth and history of physical intimate partner violence have been associated with nonuse of FP methods [12, 17, 25, 26]. Women’s reasons for not using FP include wanting to talk with their partner first [23], waiting until their current baby is older [23], waiting until their menses return [27], and fearing side effects and health risks [28].

### Provider predictors of FP use

Health providers’ perceptions and biases may influence provision of services and subsequent likelihood of client FP uptake. Providers may counsel clients against using certain methods or impose medically unfounded barriers to FP methods based on perceived client characteristics including age, partner consent, marital status, and parity [7, 23, 29, 30]. Providers’ personal biases have been found to be more commonly imposed for long-term or permanent methods [29], for condoms and pills depending on client age [29] and parity [7], and among providers in private facilities [8] or those with less FP in-service training [7, 29]. In contrast, provider willingness to share personal experiences of FP use [31] and perception of client characteristics, such as education and ability to understand FP options [29], may be positively associated with provider engagement in more directed FP counseling, and may result in greater client satisfaction with  services [32].

### Reproductive health in Kigoma region Tanzania

In 2016, Kigoma Region’s TFR was 6.5 births per woman, the modern FP prevalence rate among women in union was 20%, and the unmet need for FP among women in union was 36% [33]. There are several national Ministry of Health, Community Development, Gender, Elderly, and Children (MoHCDGEC) ongoing efforts to improve maternal health in Tanzania that have particularly targeted Kigoma Region. These include the *National Roadmap Strategic Plan to Accelerate Reduction of Maternal, Newborn and Child Deaths in Tanzania 2008–2015*; *the Big Results Now* (BRN) initiative; and Wazazi Nipendeni (“Parents Love Me”), a mobile health initiative that includes FP and safe motherhood text messages. Since 2006, the *Project to Reduce Maternal Deaths in Tanzania* has worked in the region with the aim of decreasing maternal mortality through strengthening emergency obstetric and neonatal care and improving access to and use of FP. As part of monitoring and evaluation activities conducted for the Bloomberg Philanthropies-funded *Project to Reduce Maternal Deaths in Tanzania*, the Centers for Disease Control and Prevention (CDC) has been providing technical assistance to document the integration of FP into health facilities providing maternity care in Kigoma.

### Gaps in literature

Previous studies have explored outcomes of FP counseling and service provision into non-FP services or have described the influence of provider or client characteristics on FP uptake. However, the literature reflects inconsistent associations between receipt of FP information and uptake in methods, and further inconsistencies in the role of client and provider factors in FP integration. Furthermore, there are no published studies on the influence of both client and provider characteristics as predictors of FP integration. This study helps to fill these gaps in the evidence by considering both client and provider characteristics associated with receipt of FP information and modern method among non-FP clients in Kigoma, Tanzania. This information will provide the Tanzanian MoHCDGEC, as well as other stakeholders, with evidence needed to make informed decisions about programmatic and policy implementation for FP integration into maternal and reproductive health services.

## Methods

### Study setting and design

Kigoma Region covers 45,066 km^2^ and is located in the northwestern Tanzania. In 2012, the region had a population of 2,127,930; approximately 83% of the population is designated as rural with farming as the primary economic activity. Nine out of 10 people in Kigoma have attained a primary school education and 76% of the adult population is literate. [34]

We conducted cross-sectional surveys consisting of facility-based client exit interviews and provider interviews across 61 facilities (6 hospitals, 25 health centers, and 30 dispensaries) in Kigoma Region, Tanzania from April 30 to July 1, 2016.

### Sampling and data collection

#### Facility sampling

All governmental and private hospitals (*n* = 6) and health centers (excluding those in refugee camp settings) (*n* = 25) in Kigoma Region were included. A sample of 30 dispensaries (of the 198 in the region providing delivery services) was selected to maximize geographic distribution and meet the following inclusion criteria: 1) have an estimated 180 or more births per year; 2) have two or more onsite health providers; 3) be a site for national or project partner facility improvements, and 4) refer patients to one of the 25 health centers.

#### Provider and client sampling

Convenience sampling was used to enroll providers and clients within selected facilities; all providers and clients providing/seeking care on study days were invited to participate if they met inclusion criteria. All types of providers who routinely conducted labor and delivery, pregnancy loss (PL; defined as spontaneous or induced abortion), FP, or postnatal care services were eligible, except for medical doctors and specialists who had limited numbers and availability. Clients were eligible if they were 15 to 49 years of age and received delivery, PL, and routine out-patient (FP, antenatal care [ANC], well-baby, and other) care services at the facility. Clients were excluded if they were younger than 15 or older than 49 years of age, delivered at home or on the way to the facility, had a cesarean section delivery, or experienced a stillbirth or neonatal death.

We determined that 189 provider interviews were sufficient to detect a 5% relative mean change in key variables of interest related to their knowledge and practice with 90% power and an alpha of 0.05. A sample of 908 client interviews was needed to detect a 15% absolute difference in the variables of interest with 90% power and an alpha of 0.05 (assuming a 50% reference proportion). We aimed to interview between two and six delivery and outpatient clients per provider per care type to reduce provider-specific bias; this selection rule was not applied to PL interviews due to the limited availability of PL clients and providers.

#### Interview procedures

Questionnaires were pre-tested in January 2016. Final questionnaires were translated from English to Swahili and back-translated to English. Providers were interviewed if they met inclusion criteria and consented to participate. Their clients were approached for participation as they exited care services; clients were asked which care service they received and the corresponding interview guide was administered. All interviews were administered face-to-face by an interviewer in Swahili.

#### Study tools

For client exit interviews, three separate questionnaires were used that were tailored to client service provision type. Questionnaires captured sociodemographic characteristics, perceptions of and satisfaction with services, facility experiences, receipt of FP information and uptake by method, and pregnancy history and intention. Providers completed a questionnaire and knowledge test designed to capture information about provider demographic characteristics, education, training, clinical knowledge, and practices related to labor and delivery care, newborn care, PL, and FP care.

### Outcome variables

In this analysis, we examined two binary outcome variables based on women’s self-report: 1) *Receipt of FP information* (0, did not receive FP information; 1, received FP information); and 2) *Receipt of a modern FP method* (pill, injection, intrauterine device [IUD], implant, male or female condom, male or female sterilization, or emergency contraception: 0, did not receive a modern FP method; 1 received a modern FP method).

### Independent variables

#### Client-level

The client-level variables of interest included:

- *Client age* (15 to 19, 20 to 29, 30 to 39, 40 to 49),
- *Delivery care was the primary service sought* (yes, no),
- *PL care was the primary service sought* (yes, no),
- *Well-baby care was the primary service sought* (yes, no; [immunization, growth]),
- *Other routine services was the primary service sought* (yes, no; [postnatal care, HIV testing and management, STI]),
- *Literacy* (able to read and write, able to read or write/neither read nor write),
- *Highest education attended* (no formal education, primary, secondary, university),
- *Number of prior pregnancies* (zero to three, four or more),
- *Number of live births* (zero, one to two, three to five, six or more)*,*
- *Marital status* (not in union, in union),
- *Frequency of attendance at religious services* (less than once a week, once a week or more often),
- *Wealth* (low, middle, high wealth),
- *Has heard family planning messages in the last three months* (yes, no),
- *Desired timing of future childbearing* (wants a child in the next two years, wants a child in more than two years/does not want),
- *FP discussion with partner* (no discussion, has discussed FP with partner),
- *FP decision-making* (does not make own FP decisions, able to make own FP decisions),
- *FP information index* (received information about zero or one FP method, received information about two or more FP methods; variable only used for the *Receipt of a modern FP method* model), and
- *FP information source index* (received FP information from zero or one source, received FP information from two or more sources; variable only used for the *Receipt of a modern FP method* model).

The variable *Wealth* was developed using principal components analysis (PCA) where household assets and characteristics were weighted based on their contribution to the first component and summed to create an index [35]. Each household was given a wealth index score categorized into terciles representing low, middle, and high levels of relative household wealth.

#### Provider-level

The provider-level variables of interest included:

- *Provider age* (younger than the mean of 38 years of age, 38 years or more),
- *Sex* (male, female),
- *Highest education completed* (primary, secondary, university),
- *Cadre* (clinicians [Assistant Medical Officers/Clinical Officers/Assistant Clinical Officers/Clinical Assistants], nurses/midwives [Nurse Officers/Assistant Nurse Officers/Registered Nurses/Midwives/Enrolled Nurses], other staff [Medical Attendants/Maternal and Child Health Aides]),
- *Years in cadre* (less than the mean of 11 years, 11 or more years),
- *Years at the facility* (less than the mean of eight years, eight years or more),
- *Work hours per week* (less than the mean of 56 h per week, 56 or more hours per week),
- *Job satisfaction* (a little/very satisfied, a little/very dissatisfied/neither satisfied nor dissatisfied),
- *Perceptions of fairness in pay* (feels he/she is not paid fairly, feels paid fairly),
- *Perceptions of adequacy of training* (feels training is inadequate for job duties, feels training is adequate),
- *Regularly provides FP services* (yes, no),
- *FP bias* (reports no bias against providing FP service, bias reported on one or more client characteristics^1^),
- *FP recent-training PCA score* (low [below mean], high [mean or higher]),
- *FP ever-training PCA score* (low [below mean], high [mean or higher]),
- *FP practice PCA score* (low [below mean], high [mean or higher]),
- *Perceptions of helpfulness of in-service training* (feels in-service training has not been helpful, feels in-service training has been helpful),
- *Electronic mentoring opportunities* (no access to e-learning/ emergency call system/ teleconference, has access to one or more electronic mentoring activities).

The variables for *FP recent-training PCA score* (FP training in 2015 or in the first half of 2016), *FP ever-training PCA score* (FP pre- or in-service training at any time), and *FP practice PCA score* (current provision of FP services) were developed with PCA using 14 items that cover a range of FP topics.^2^ Elements for recent-training, ever-training, and current practice were weighted based on their contribution to the first principal component and summed to create three separate indices.

## Analytic approach

Client and provider data were linked. Due to the focus of this study on FP integration into non-FP services, FP clients (*n* = 214), current FP users (*n* = 128), and providers who only gave care to FP clients (*n* = 38), were excluded from analyses. ANC (*n* = 225) and other pregnant clients (*n* = 40) were also excluded due to lack of data on desire for future childbearing and FP decision-making.

Data analyses were conducted using Stata 14. Bivariate analyses were conducted to identify client and provider variables associated with the outcome variables of interest. Client and provider variables with an unadjusted association with the outcomes of interest at a level of *p* < 0.10 were included in multivariate modeling. A multilevel mixed-effects logistic regression model was used to examine the effects of patient and provider characteristics on either receipt of FP information or receipt of a modern FP method, with random intercepts for each provider. A *p*-value of less than 0.05 was considered statistically significant for multilevel models. We included facility-clustered standard errors to account for unobserved heterogeneity within facilities.

## Results

From April 30–July 1, 2016, 2155 female clients (delivery *n* = 960, PL *n* = 230, outpatient *n* = 965 [inclusive of antenatal, postnatal care, HIV testing and management, STI, and other) and 361 providers were interviewed. Following removal of data from non-linked clients and providers, FP clients, pregnant clients, and current FP users, data from 1505 clients (primary reasons for visit: delivery *n* = 935, PL *n* = 229, well-baby *n* = 272, other routine services *n* = 69) and 330 providers (clinicians *n* = 69, nurses/midwives *n* = 176, other staff *n* = 85) were used in the analysis.

### Client and provider characteristics

Descriptive characteristics of the clients included in the analyses are displayed in Table 1. The majority of clients were in a union (90.4%), had attended religious services at least weekly (85.8%), were able to read and write (71.1%), and had attended primary school education (65.8%). About half of clients were 20 to 29 years of age (49.8%) and received care at a health center (48.9%). Two-thirds of clients came to the facility for the purpose of delivery services (62.1%), while less than one-fifth came for the purpose of well-baby (18.1%), PL (15.2%), and other services (4.6%). Whereas 59.4% of clients reported having a FP discussion with their partner, less than one-fifth of clients (17.9%) reported feeling they are able to make their own FP decisions.

**Table 1:** Characteristics of Women Included in the Family Planning Integration Study Sample—Kigoma Region, Tanzania, April to July 2016 (n = 1505)

	Women, n (%)	95% CI
Age in years		
15–19	214 (14.2)	12.5–16.0
20–29	750 (49.8)	47.3–52.4
30–39	427 (28.4)	26.1–30.7
40–49	93 (6.2)	5.0–7.4
Don’t know	21 (1.4)	0.8–2.0
Delivery care was primary service sought		
Yes	935 (62.1)	59.7–64.6
No	570 (37.9)	35.5–40.4
Well-baby care was primary service sought		
Yes	272 (18.1)	20.5–24.8
No	1233 (81.9)	79.9–83.8
Pregnancy loss (PL) care was primary service sought		
Yes	229 (15.2)	13.4–17.0
No	1276 (84.8)	82.9–86.5
Other routine services (postnatal care, HIV testing and management, STI, other) was primary service sought		
Yes	69 (4.6)	3.5–5.6
No	1436 (95.4)	94.2–96.4
Facility type		
Hospital	421 (28.0)	25.7–30.2
Health center	736 (48.9)	46.4–51.4
Dispensary	348 (23.1)	21.0–25.3
Highest education attended		
No education	325 (21.6)	19.5–23.7
Primary	990 (65.8)	63.4–68.2
Secondary or higher	190 (12.6)	10.9–14.3
Literacy		
Can read and write	1070 (71.1)	68.8–73.4
Cannot read or write	360 (23.9)	21.7–26.1
Can either read or write, but not both	64 (4.3)	3.2–5.3
Missing or refused	11 (0.7)	0.3–1.2
Wealth		
Low wealth	478 (31.8)	29.4–34.1
Middle wealth	514 (34.2)	31.8–36.6
High wealth	513 (34.1)	31.7–36.5
Number of live births		
None	43 (2.9)	2.0–3.7
1 to 2	662 (44.0)	41.5–46.5
3 to 5	504 (33.5)	31.1–35.9
6+	293 (19.5)	17.5–21.5
Missing	3 (0.2)	0.0–0.4
Number of prior pregnancies		
0 to 3	832 (55.3)	52.8–57.8
4+	673 (44.7)	42.2–47.2
Marital status		
In a union	1361 (90.4)	88.8–91.8
Not in a union	144 (9.6)	8.2–11.2
Has had FP discussion with partner		
Yes	894 (59.4)	56.9–61.9
No or don’t know	603 (40.1)	37.6–42.5
Missing	8 (0.5)	0.2–0.9
Able to make own FP decisions		
Yes	270 (17.9)	16.0–19.9
No or don’t know	1231 (81.8)	79.8–83.7
Missing	4 (0.3)	0.0–0.5
Received FP information during non-FP visit		
Yes	778 (51.7)	49.2–54.2
No	727 (48.3)	45.8–50.8
Number of FP methods information was received about during non-FP visit		
0 or 1 FP method	798 (53.0)	50.5–55.5
2 or more FP methods	707 (47.0)	44.5–49.5
Modern FP methods information received about among women who reported receiving information about 1 or more methods (*n* = 778)^a^		
Injection	623 (80.1)	77.1–82.7
Implant	612 (78.7)	75.6–81.4
Intrauterine device	549 (70.6)	67.3–73.7
Pills	528 (67.9)	64.5–71.1
Male condom	262 (33.7)	30.4–37.1
Female condom	117 (15.0)	12.7–17.7
Tubal ligation	100 (12.9)	10.7–15.4
Vasectomy	28 (3.6)	2.5–5.2
Emergency Contraception	25 (3.2)	2.2–4.7
Number of sources FP information was received from during non-FP visit		
0 or 1 source of FP information	1349 (89.6)	88.0–91.1
2 or more sources of FP information	155 (10.3)	8.9–11.9
Missing	1 (0.1)	0.0–0.5
Sources that FP information received from among women who reported receiving information from 1 or more sources (*n* = 777)^a^		
Provider spoke to client	766 (98.5)	97.3–99.1
FP educational talk	89 (11.3)	9.3–13.8
FP educational flyer	67 (8.6)	6.8–10.8
Client asked provider	50 (6.3)	4.8–8.3
FP educational video	10 (1.3)	0.7–2.4
Received modern FP method during non-FP visit		
No	1378 (91.6)	90.0–92.9
Yes	127 (8.4)	7.1–10.0
Modern FP method received among women who reported receiving a modern method (*n* = 127)^a^		
Injection	43 (33.9)	26.1–42.6
Implant	39 (30.7)	23.2–39.4
Male condom	18 (14.2)	9.1–21.5
Intrauterine device	14 (11.0)	6.6–17.9
Pill	9 (7.1)	3.7–13.2
Tubal ligation	3 (2.4)	0.8–7.2
Female condom	1 (0.8)	0.1–5.5
Heard FP messages from any source in last 3 months		
Yes	555 (36.9)	34.4–39.3
No or don’t know	950 (63.1)	60.7–65.6
Desired timing of future childbearing		
1–2 years	287 (19.1)	17.8–21.1
More than 2 years	852 (56.6)	54.1–59.1
Does not want	288 (19.1)	17.1–21.1
Don’t know or refused	78 (5.2)	4.1–6.3
Frequency of attendance at religious services		
Attends at least weekly	1291 (85.8)	84.0–87.5
Attends less often than weekly	214 (14.2)	12.5–16.0

^a^Denotes sum greater than 100% due to multiple methods/sources reported by some clients

Descriptive characteristics of the providers included in the analyses are displayed in Table 2. The majority of providers included in the study were female (63.3%), college/university educated (66.7%), worked in a health center (55.2%), and were in the nurse/midwife cadre (53.3%). About two-thirds of providers have worked for more than 10 years in the cadre (61.5%) and more than 7 years at their current facility (70%). Providers worked an average of 54 h per week, with 57.9% working more than this average. Less than half of providers were a little or very satisfied with their job (44.9%), and less than one-fifth of providers reported feeling they were paid fairly for their job duties (17.0%). Three-quarters routinely provided FP services (75.5%), and nearly two-thirds of providers displayed at least one type of FP bias related to age, parental consent, spousal consent, or parity (62.3%).

**Table 2:** Characteristics of Providers included in the Family Planning Integration Study Sample—Kigoma Region, Tanzania, April to July 2016 (*n* = 330)

	Providers, n (%)	95% CI
Age in years		
20–29	125 (37.9)	32.6–43.1
30–39	54 (16.4)	12.4–20.4
40–49	74 (22.4)	17.9–26.9
50+	77 (23.3)	18.7–27.9
Sex		
Female	209 (63.3)	58.1–68.6
Male	121 (36.7)	31.4–41.9
Highest education completed		
Primary	15 (4.6)	2.3–6.8
Secondary	95 (28.8)	23.9–33.7
College/university	220 (66.7)	61.6–71.8
Cadre		
Clinician	69 (20.9)	16.5–25.3
Nurse/midwife	176 (53.3)	47.9–58.7
Other staff	85 (25.7)	21.0–30.5
Years in cadre		
0 to 10 years	127 (38.5)	33.2–43.8
11+ years	203 (61.5)	56.2–66.8
Years at the facility		
0 to 7 years	99 (30.0)	25.0–35.0
8+ years	231 (70.0)	65.0–75.0
Facility type		
Hospital	82 (24.9)	20.2–29.5
Health center	182 (55.2)	49.8–60.5
Dispensary	66 (20.0)	15.7–24.3
Work hours per week		
54+ hours per week	191 (57.9)	52.5–63.2
Less than 54 h per week	139 (42.1)	36.8–47.5
Routinely provides FP services		
Yes	249 (75.5)	70.8–80.1
No	81 (24.6)	19.9–29.2
FP ever-training PCA score^a^		
Low	132 (40.0)	34.7–45.3
High	198 (60.0)	54.7–65.3
FP recent-training PCA Score^a^		
Low	227 (68.8)	63.8–73.8
High	103 (31.2)	26.2–36.2
FP Practice PCA Score^a^		
Low	131 (39.7)	34.4–45.0
High	199 (60.3)	55.0–65.6
Access to electronic mentoring opportunities		
No access	165 (50.0)	44.6–55.4
Access to at least 1 type (emergency call system, e-learning, teleconference)	165 (50.0)	44.6–55.4
Perception in-service training has helped job performance		
Yes	285 (86.4)	82.6–90.1
No	45 (13.6)	9.9–17.4
Job Satisfaction		
Very or a little satisfied	148 (44.9)	39.5–50.2
Neutral, a little dissatisfied or very dissatisfied	182 (55.2)	49.8–60.5
Perception paid fairly for job duties		
Yes	56 (17.0)	12.9–21.0
No	274 (83.0)	79.0–87.1
Perception of adequacy of training for job duties		
Yes	227 (68.8)	63.8–73.8
No	103 (31.2)	26.2–36.2
Displays at least 1 FP bias^b^		
Yes	207 (62.7)	57.5–68.0
No	123 (37.3)	32.0–42.5

^a^Providers were asked if they had ever received training in, had recently received training in (in the last 18 months), and if they had provided the service in the last 3 months related to the following 14 FP items: 1) counsel women about FP and contraception; 2) give an injectable; 3) insert an IU(C)D; 4) remove an IU(C)D; 5) insert an implant; 6) remove an implant; 7) perform a tubal ligation; 8) perform a vasectomy; 9) provide counseling on FP options; 10) provide counseling on the efficacy of FP methods; 11) provide counseling on the potential side effects of FP methods; 12) provide counseling on the potential warning signs of FP methods; 13) provide clinical management of FP side effects; and 14) provide FP for HIV positive women

^b^Providers were asked if they decide whether or not to provide FP services to clients based on one or more of the following client characteristics: marital status/parental consent/partner consent/age/parity

### Receipt of FP information

#### Descriptive characteristics for receipt of FP information

**Fig. 1:**
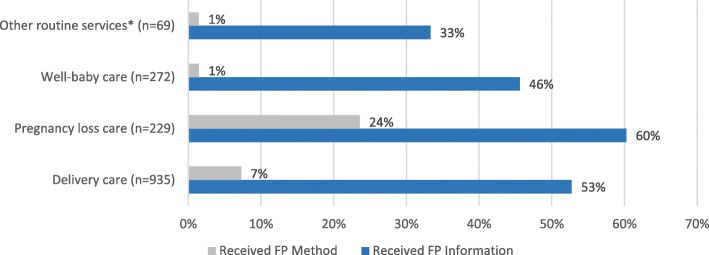
Receipt of Family Planning Information and a Modern Method by Care Type—Kigoma Region, Tanzania, April to July 2016 (*n* = 1505). *Other routine services included clients who sought postnatal care, HIV testing and treatment, sexually transmitted infection (STI) care, and others.

About half of clients reported receiving FP information during their non-FP visit (51.7%). Among those who reported receiving FP information, the provider speaking with the client was the most common source of FP information (98.5%). The most commonly discussed methods were injection (80.1%), implant (78.7%), and intrauterine device (70.6%) (Table 1). More than half of clients reported wanting to wait for more than two years before having another child (56.6%), and 19.1% of clients reported not desiring to have any more children. More than half of PL (60.3%) and delivery (52.7%) care clients reported receiving FP information, compared to 45.6% of well-baby care and 33.3% of other routine services clients (Fig. 1).

#### Bivariate analyses

Results of bivariate analyses for *Receipt of FP information* are displayed in the Appendix. Client covariates with a significant positive association with receipt of FP information included *Client age*, *PL care was the primary service sought*, *Highest education attended*, *Wealth*, *Number of prior pregnancies*, *Has had FP discussion with partner,* and *Heard FP messages in the last 3 months*. Client covariates with a significant negative association with receipt of FP information included *Other routine services was the primary service sought*, *Frequency of attendance at religious services*, and *Desired timing of future childbearing*.

Provider covariates with a significant positive association with receipt of FP information included *Provider highest education completed*, *Routinely provides FP services*, *FP Ever-training PCA score*, *FP Recent-training PCA score*, *FP Practice PCA score*, *Perception in-service training has helped job performance, and Access to electronic mentoring opportunities*. *Hours worked per week* was the only provider covariate with a significant negative association with receipt of FP information.

#### Multilevel analyses

Results of multilevel, mixed-effects logistic regression analyses for *Receipt of FP information* are shown in Table 3. The odds of receiving FP information were higher for PL clients vs those seeking any other types of care (aOR 1.97, 95% CI 1.01–3.84), for those with four or more vs fewer pregnancies (aOR 1.78, 95% CI 1.21–2.60), and for those who had, vs had not, had a FP discussion with their partner (aOR 1.73, 95% CI 1.33–2.25).

**Table 3:** Multilevel Mixed-Effects Logistic Regression Analysis for Receipt of Family Planning (FP) Information—Kigoma Region, Tanzania, April to July 2016 (Clients *n* = 1497, Providers *n* = 330)

	Adjusted OR (95% CI)	*p*-value
FIXED EFFECTS - CLIENT LEVEL VARIABLES		
Client age in years		
15–19 (reference)		
20–29	0.93 (0.66–1.33)	0.707
30–39	0.61 (0.37–1.03)	0.063
40–49	0.50 (0.26–0.97)	0.039
Don’t know	4.11 (0.95–17.76)	0.058
Highest education attended		
No education		
Primary	1.25 (0.87–1.79)	0.230
Secondary	1.53 (0.93–2.51)	0.093
College or university	1.03 (0.39–2.74)	0.947
Pregnancy loss (PL) care was primary service sought		
No (reference)		
Yes	1.97 (1.01–3.84)	0.048
Other routine services was primary service sought		
No (reference)		
Yes	0.43 (0.18–1.03)	0.058
Frequency of religious service attendance		
Less often than weekly (reference)		
Weekly or more often	0.61 (0.44–0.85)	0.003
Wealth		
Low wealth (reference)		
Middle wealth	1.24 (0.82–1.88)	0.300
High wealth	1.27 (0.81–1.97)	0.294
Number of prior pregnancies		
0 to 3 (reference)		
4+	1.78 (1.21–2.60)	0.003
Desired timing of future childbearing		
More than 2 years, or does not want (reference)		
1 to 2 years	0.73 (0.51–1.04)	0.079
Heard FP messages in last three months		
No (reference)		
Yes	1.21 (0.88–1.67)	0.233
Has had FP discussion with partner		
No or don’t know (reference)		
Yes	1.73 (1.33–2.25)	0.000
FIXED EFFECTS - PROVIDER-LEVEL VARIABLES		
Highest education completed		
Primary (reference)		
Secondary	1.25 (0.60–2.63)	0.552
College/University	2.03 (0.97–4.25)	0.060
Work hours per week		
Less than 54 h per week (reference)		
54+ hours per week	0.68 (0.45–1.01)	0.054
Routinely provides FP services		
No (reference)		
Yes	1.45 (0.85–2.47)	0.170
FP ever-training PCA score^a^		
Low (reference)		
High	1.01 (0.57–1.79)	0.970
FP recent-training PCA score^a^		
Low (reference)		
High	1.58 (1.11–2.26)	0.012
FP Practice PCA Score^a^		
Low (reference)		
High	0.86 (0.48–1.53)	0.606
Access to electronic mentoring opportunities		
No access (reference)		
Access to at least 1 type (emergency call system, e-learning, teleconference)	1.38 (0.85–2.23)	0.191
Perception in-service training has helped job performance		
No (reference)		
Yes	2.27 (1.35–3.84)	0.002
RANDOM EFFECTS		
Provider-level variance (SE)	1.21 (0.28)	
Provider-level variance partition coefficient	0.27 (0.19–0.37)	
Level 1 units	1497	
Level 2 units	330	
Log likelihood	− 921.18412	

^a^Providers were asked if they had ever received training in, had recently received training in (in the last 18 months), and if they had provided the service in the last 3 months related to the following 14 FP items: 1) counsel women about FP and contraception; 2) give an injectable; 3) insert an IU(C)D; 4) remove an IU(C)D; 5) insert an implant; 6) remove an implant; 7) perform a tubal ligation; 8) perform a vasectomy; 9) provide counseling on FP options; 10) provide counseling on the efficacy of FP methods; 11) provide counseling on the potential side effects of FP methods; 12) provide counseling on the potential warning signs of FP methods; 13) provide clinical management of FP side effects; and 14) provide FP for HIV positive women

The odds of receiving FP information were lower for clients aged 40 to 49 years vs those aged 15 to 19 years (aOR 0.50, 95% CI 0.26–0.97) and for clients who reported attendance at religious services weekly or more often vs those who reported less frequent attendance (aOR 0.61, 95% CI 0.44–0.85). Client-level variables found not to have a significant adjusted relationship with receipt of FP information include client *Other services was primary service sought, Highest education attended*, *Wealth*, *Desired timing of future childbearing*, and *Heard FP messages in last three months*.

The odds of receiving FP information were higher for clients of providers who perceived that in-service training has vs has not helped their job performance (aOR 2.27, 95% CI 1.35–3.84), and for clients of providers who had high as compared to low FP recent training scores (aOR 1.58, 95% CI 1.11–2.26). Provider-level variables found not to have a significant adjusted relationship with receipt of FP information include *Provider highest education completed*, *Work hours per week*, *Routinely provides FP services*, *FP ever-training PCA score*, *FP practice PCA score*, and *Access to electronic mentoring opportunities*.

The intraclass correlation (ICC) describes the proportion of the total variance that can attributed to the hierarchal grouping by the provider variable. Net of all predictors included in the final FP information model, about one-quarter of the total variance (ICC = 0.27) occurred between providers.

### Receipt of a modern FP method

#### Descriptive characteristics for receipt of a modern FP method

**Fig. 2:**
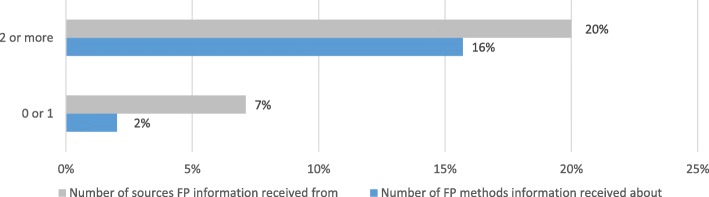
Percentage of Clients Who Received a Modern Family Planning Method by Number of Methods Information was Received about and Number of Sources Information was Received from—Kigoma Region, Tanzania, April to July 2016 (n = 1505)

Less than one in 10 clients (8.4%) reported receiving a modern FP method; among those who reported receiving a modern method, injection (33.9%), implant (30.7%), and male condoms (14.2%) were the most common methods received (Table 1). About one-quarter of PL (23.6%) and 7.3% of delivery care clients reported receiving a modern method, while only 1.5% of well-baby care and other routine services clients reported receiving a method (Fig. 1). Sixteen percent (15.7%) of clients who received information on two or more FP methods reported receiving a modern FP method at their care visit, while only 2.0% of clients who received information on zero or one method reported this (Fig. 2). Similarly, one-fifth of clients (20.0%) who received FP information from two or more sources reported receiving a modern FP method, while only 7.1% of clients who received FP information from zero or one source reported receiving a method (Fig. 2).

#### Bivariate analyses

Results of bivariate analyses for *Receipt of a modern FP method* are displayed in the Appendix. The client covariates with a significant positive association with receipt of a modern contraceptive method included *PL care was the primary service sought*, *Client highest education attended*, *Literacy*, *Wealth, Number of live births*, *Has had FP discussion with partner*, *Able to make own FP decisions*, *Number of FP methods received information about*, and *Number of sources for FP information*. Client covariates with a significant negative association with receipt of a modern contraceptive method included *Well-baby care was the primary service sought*, *Other routine services was the primary service sought, Marital status*, and *Desired timing of future childbearing*.

Provider covariates with a significant positive association with receipt of a modern contraceptive method included *FP Practice PCA score* and *Access to electronic mentoring opportunities*. Provider covariates with a significant negative association with receipt of a modern contraceptive method included *Provider age*, *Cadre, and Work hours per week*.

#### Multilevel analyses

Results of multilevel, mixed-effects logistic regression analyses for receipt of a modern FP method are shown in Table 4. The odds of receiving a modern FP method were higher for clients who received information on two or more vs fewer methods (aOR 7.13, 95% CI 2.13–23.80), for those who had a FP discussion with their partner (aOR 5.87, 95% CI 2.81–12.28), for clients seeking PL care vs those seeking all other types of care (aOR 4.08, 95% CI 1.97–8.44), for those with zero vs one or more live births (aOR 3.92, 95% CI 1.40–10.94), for those who reported they are able to make their own FP decisions (aOR 3.17, 95% CI 1.55–6.49), for clients who received FP information from two or more vs fewer sources (aOR 3.12, 95% CI 1.40–6.92), and for those in the middle and high wealth terciles vs low wealth tercile (aOR 1.99, 95% CI 1.11–3.56; aOR 2.30, 95% CI 1.30–4.07, respectively).

**Table 4:** Multilevel Mixed Effects Logistic Regression Analysis for Receipt of a Modern Family Planning (FP) Method—Kigoma Region, Tanzania, April to July 2016 (Clients *n* = 1482, Providers *n* = 330)

	Adjusted OR (95% CI)	*p*-value
FIXED EFFECTS - CLIENT LEVEL VARIABLES		
Highest education attended		
No education (reference)		
Primary	0.43 (0.17–1.13)	0.088
Secondary or higher	0.85 (0.24–3.03)	0.799
University	1.39 (0.16–11.87)	0.765
Literacy		
Cannot read or write, or can do one (reference)		
read and write	1.89 (0.72–4.91)	0.193
Pregnancy loss (PL) care was primary service sought		
No (reference)		
Yes	4.08 (1.97–8.44)	0.000
Other services was primary service sought		
No (reference)		
Yes	0.08 (0.01–0.87)	0.038
Well-baby care was primary service sought		
No (reference)		
Yes	0.14 (0.04–0.54)	0.004
Marital status		
Not in a union (reference)		
In a union	0.41 (0.18–0.93)	0.034
Wealth		
Low wealth (reference)		
Middle wealth	1.99 (1.11–3.56)	0.021
Highest wealth	2.30 (1.30–4.07)	0.004
Live births		
1 or more (reference)		
None	3.92 (1.40–10.94)	0.009
Has had FP discussion with partner		
No or don’t know (reference)		
Yes	5.87 (2.81–12.28)	0.000
Can make own FP decisions		
No or don’t know (reference)		
Yes	3.17 (1.55–6.49)	0.002
Number of FP methods received information about during non-FP visit		
0 to 1 (reference)		
2 or more	7.13 (2.13–23.80)	0.001
Number of sources FP information received from during non-FP visit		
0 to 1 (reference)		
2 or more	3.12 (1.40–6.92)	0.005
Desired timing of future childbearing		
More than 2 years or does not want (reference)		
1–2 years	0.47 (0.20–1.13)	0.090
FIXED EFFECTS - PROVIDER-LEVEL VARIABLES		
Age in years		
38 years or younger (reference)		
39+ years	0.77 (0.37–1.62)	0.498
Cadre		
Clinician (reference)		
Nurse/midwife	0.81 (0.41–1.59)	0.535
Other staff	0.90 (0.35–2.30)	0.824
Hours worked per week		
55 h or less per week (reference)		
56+ hours	0.63 (0.35–1.15)	0.132
Access to electronic mentoring opportunities		
No access (reference)		
Access to at least 1 type (emergency call system, e-learning, teleconference)	1.27 (0.57–2.82)	0.562
FP Practice PCA Score^a^		
Low (reference)		
High	1.14 (0.60–2.17)	0.685
RANDOM EFFECTS		
Provider-level variance (SE)	1.39 (0.71)	
Provider-level variance partition coefficient	0.30 (0.13–0.53)	
Level 1 units	1482	
Level 2 units	330	
Log likelihood	− 292.07447	

^a^Providers were asked if they had provided the service in the last 3 months related to the following 14 FP items: 1) counsel women about FP and contraception; 2) give an injectable; 3) insert an IU(C)D; 4) remove an IU(C)D; 5) insert an implant; 6) remove an implant; 7) perform a tubal ligation; 8) perform a vasectomy; 9) provide counseling on FP options; 10) provide counseling on the efficacy of FP methods; 11) provide counseling on the potential side effects of FP methods; 12) provide counseling on the potential warning signs of FP methods; 13) provide clinical management of FP side effects; and 14) provide FP for HIV positive women

Well-baby care clients, Other services clients, and married clients had significantly lower odds of receiving a FP method compared to clients seeking any other type of services and unmarried clients (aOR 0.14, 95% CI 0.04–0.54; aOR 0.08, 95% CI 0.01–0.87; aOR 0.41, 95% CI 0.18–0.93, respectively). Client-level variables found not to have a significant adjusted relationship with receipt of a FP method include *Highest education attended*, *Literacy*, and *Desired timing of future childbearing*.

None of the provider-level variables were found to have a significant adjusted relationship with receipt of a modern FP method by a client. Net of all predictor variables included in the FP method model, nearly 30% of the total variance (ICC 0.30) occurred between providers.

## Discussion

Integrating FP service provision into other types of healthcare visits can provide an important opportunity to reduce the unmet need for FP and ultimately lower maternal and neonatal mortality. We sought to understand the factors that predicted receipt of FP information and a modern FP method among women attending maternal, child, and reproductive health care services in Kigoma, Tanzania. The results demonstrate the association between the receipt of FP information with both the client and provider characteristics, while receipt of a modern FP method is associated with only client factors. This information will help inform FP training and programming in Kigoma, Tanzania.

First, level of FP integration is not consistent across non-FP care types. Unlike results from a study in Senegal, which found low receipt of FP information among PL clients compared to clients of other care types [16], our study found that women who sought PL services had nearly two times greater odds of receiving FP information and more than four times greater odds of receiving a modern method compared to women seeking other types of services. In contrast, women who sought well-baby care services and other routine care services had 86 and 92% lower odds of receiving a modern FP method, respectively.

Women’s self-efficacy, assessed with questions about past FP discussions with partner and ability to make one’s own FP decisions, was strongly associated with receipt of information and modern FP methods. Women who reported past FP discussions with their partner as compared with those who did not had nearly two times greater odds of receiving FP information and more than five times greater odds of receiving a FP method. In addition, women who reported the ability to make their own FP decisions had three times greater odds of receiving a method compared to women unable to make their own decisions. These results align with previous studies in India, Senegal, and Turkey, which also found that that partner communication [16, 25] and self-efficacy [20] are positively associated with FP uptake. These findings suggest that women who have had conversations with their FP partners and/or feel they can make their own decisions may be initiating FP conversations with providers, thereby driving the receipt of information and a method during a care visit.

The quantity of FP information received at the visit, including the number of methods information was received about and the number of sources information came from, was strongly associated with FP method receipt. Women who received FP information about two or more methods had seven times greater odds of receiving a contraceptive method compared to women who received FP information about one or zero methods. Similarly, women who received FP information from two or more sources had nearly three times greater odds of receiving a method compared to women who received FP information from one or zero sources. These findings may inform elements of counseling in clinical practice.

Additional demographic characteristics, including parity, marital status, religiosity, and wealth, were also associated with receipt of FP information and modern FP methods. Women with four or more live births had nearly two times greater odds of receiving FP information compared to women with three or fewer live births. One possible explanation for this is providers’ perceptions of women’s eligibility for receiving methods. A study on provider-imposed barriers to family planning in Kenya found that women’s parity influenced providers’ method offerings; specifically, providers reported to impose FP method restrictions on women without children [7]. However, we found that women reporting having no children born alive had nearly four times greater odds of receiving a FP method. This finding may represent a desire to postpone childbearing. Specifically, among this sub-group of clients with no live births, a comparatively higher proportion were teenagers (38.1% vs 13.6%), unwed (25.6% vs 8.9%), and seeking PL care (93.0% vs 13.0%) compared to those with live births. We also found that women with no live births were not more likely to receive FP information, suggesting that this sub-group of clients were driving the requests for receipt of FP methods.

Women who reported more frequent attendance at religious services had 39% lower odds of receiving FP information compared to women with less frequent attendance. This finding suggests that religiosity either influences women’s request for FP information or influences provider’s perception of client interest in FP. Women in the middle and high wealth categories had two and two and a half times greater odds of receiving a FP method, respectively. This finding aligns with previous findings that poorer women receiving FP counseling and education have lower uptake of FP methods [12]. Given that FP is available at no cost to women in Tanzania, this difference may reflect an inverse relationship between future fertility preferences and wealth. Alternatively, this finding might suggest a provider’s bias in offering modern methods to wealthier clients.

Recent in-service training and positive perceptions of in-service training as a whole among providers were important factors for client receipt of FP information. Other studies have found that a greater proportion of providers without in-service FP training imposed parity, age, marital, and other restrictions on provision of FP methods [7, 29, 36, 37]. In our study, clients of providers who had recent in-service training on a higher number of FP topics had one and a half times greater odds of receiving FP information at their visit compared to clients of providers who had recent in-service training on a lower number of FP topics. Similarly, clients of providers who reported that in-service training has helped their job performance had more than two times greater odds of receiving FP information compared clients of providers who reported in-service training has not helped. While we found that provider factors were important for client’s receipt of FP information, provider factors were not predictive of client’s receipt of a FP method. This suggests that client’s desire for adopting a modern method expressed during non-FP visits may be the primary driver of receiving FP methods.

Findings from this study corroborate evidence that women’s self-efficacy in FP decision-making [20], women’s specific demographic characteristics (e.g., religiosity, wealth) [12], and providers’ in-service training [7, 29] influence women’s odds of receiving a FP method. This study adds new evidence pertaining to important elements of FP counseling and receipt of a method—namely, the quantity of FP information received at a single care visit (e.g., number of methods described, number of sources of FP information). While many studies address factors associated with receipt of FP methods, this study also adds new insight into factors associated with receipt of FP information, including provider characteristics related to the timing FP in-service training, partner communication, high parity, older age, and lack of religiosity.

### Strengths and limitations

This study has several strengths. First, the study has matched client and provider data, enabling quantification of the importance of both client and provider level factors for FP integration. In addition, clients of varied care services were included in the analyses, allowing a better understanding of the strengths and limitations of FP integration by care type. Moreover, we operationalized select independent variables in ways that have not been done in previously published work. For example in the model for *Receipt of a modern FP method*, receipt of FP information was operationalized into two distinct independent variables that quantify same-visit access to information: 1) *Number of FP methods information was received about*, and 2) *Number of sources information received from*. Additionally, provider-level *FP ever-training score*, *FP recent-training score*, and *FP Practice score* variables were operationalized using multiple FP care elements to differentiate the importance of both dose and timing of training and practice to provision of FP integration services.

There are several limitations to this study that are important to consider. First, the study employed a cross-sectional design which limits our ability to make causal inferences between the independent variables and outcomes of interest. We noted wide confidence intervals for some variables in the regression analyses which likely reflect small cell sizes; these results should be interpreted with caution. Ongoing longitudinal data collection and analyses will strengthen the robustness of the potential relationships. We used convenience sampling to recruit providers and clients, limiting the generalizability of the findings within or beyond Kigoma Region. To minimize this limitation, we aimed to interview all providers and clients who met the inclusion criteria and were available for interview during the study period. Data limitations included lack of a rural versus urban variable and information about receipt of FP counselling at multiple points in time. These exclusions eliminated our ability to assess the influence of setting and counselling over time on receipt of FP information and uptake.

### Research, programmatic, and policy implications

Our findings highlight areas for future research and have clear implications for programmatic and policy work. Additional research with matched client and provider data is needed to further delineate the relative importance of client and provider factors for FP integration. Studies such as this could be replicated in other areas of Tanzania and eastern Africa, or beyond, to better understand how similar or disparate the determinants of receiving FP information and methods are across regions and countries. Future analyses would benefit from inclusion of interaction terms to better understand how specific client characteristics, such religion, age, and parity, intersect with provider perceptions and potential biases in ways that influence provision of FP services. Observational and descriptive studies are also needed to better understand logistical considerations for integration of services in facilities (e.g., location of FP providers and methods, process for receiving services either directly or through referral, other access barriers).

Increased attention is needed to better integrate FP counseling and service provision routinely into well-baby and other routine care services. Capitalizing on the year post delivery is one strategy that has been effective in other settings [15, 17, 23]. Community- and facility-based strategies are needed that encourage women to have conversations with their partners and to create service environments where women feel they can begin conversations with providers and make their own decisions to initiate FP. Based on our findings, expanding the number of FP method options and sources of information provided at single visits would also likely drive an uptake in FP use. The US-based National Campaign to Prevent Teen and Unplanned Pregnancy’s One Key Question is a program that encourages primary care providers to routinely ask women about their reproductive health goals using a simple question: would you like to become pregnant in the next year? [38] This approach, among others, could be piloted in Kigoma, and if deemed effective, incorporated into national policies.

## Conclusion

Despite much evidence establishing the salient role of FP counseling to improve reproductive health outcomes, there is less consensus about the relative impact of client or provider attributes on the receipt of FP information or a modern method. We address the question of whether including both client and provider characteristics adds predictive value when analyzing the risk of FP integration or uptake. Our findings demonstrate that both client and provider factors significantly influence FP integration. Improved understanding of the interplay between clients and providers is critical for the effective provision of FP services and should be considered in the design of reproductive health policies and future data collection exercises.

## Appendix

**Table 5:** Association between Client and Provider Characteristics and Receipt of Family Planning (FP) Information and a Modern FP Method—Kigoma Region, Tanzania, April to July 2016 (Clients *n* = 1505, Providers *n* = 330)

	Receipt of FP Information		Receipt of Modern FP Method	
	Unadjusted OR (95% CI)	*p*-value	Unadjusted OR (95% CI)	*p*-value
CLIENT LEVEL VARIABLES				
Age in years				
15–19 (reference)				
20–29	1.23 (0.85–1.77)	0.272	1.22 (0.64–2.34)	0.551
30–39	1.22 (0.82–1.81)	0.335	1.18 (0.59–2.38)	0.641
40–49	0.95 (0.51–1.75)	0.862	0.85 (0.28–2.56)	0.771
Don’t know	5.82 (1.50–22.54)	0.011	0.28 (0.03–2.90)	0.284
Delivery care was primary service sought				
No (reference)				
Yes	0.96 (0.71–1.29)	0.771	0.68 (0.43–1.08)	0.101
Well-baby care was primary service sought				
No (reference)				
Yes	0.85 (0.59–1.22)	0.377	0 .13 (0.05–0.39)	0.000
Pregnancy loss (PL) care was primary service sought				
No (reference)				
Yes	2.06 (1.32–3.20)	0.001	6.41 (3.74–11.01)	0.000
Other routine services (Postnatal, HIV, STI, other) was primary service sought				
No (reference)				
Yes	0.38 (0.20–0.75)	0.005	0.13 (0.02–1.07)	0.057
Highest education attended				
No education (reference)				
Primary	1.24 (0.91–1.70)	0.178	1.22 (0.68–2.18)	0.509
Secondary	1.55 (0.97–2.47)	0.068	4.26 (2.10–8.64)	0.000
University	1.00 (0.37–2.68)	0.997	4.38 (1.19–16.13)	0.026
Literacy				
Cannot read and write, or can read or write, but not both (reference)				
Read and write	1.15 (0.87–1.53)	0.324	2.40 (1.38–4.17)	0.002
Wealth				
Low wealth (reference)				
Middle wealth	1.31 (0.96–1.79)	0.092	2.12 (1.16–3.88)	0.015
High wealth	1.46 (1.06–2.02)	0.022	3.64 (2.02–6.58)	0.000
Number of live births				
1+ (reference)				
None	0.72 (0.34–1.51)	0.383	3.61 (1.52–8.55)	0.004
Number of prior pregnancies				
0–3 (reference)				
4+	1.38 (1.07–1.77)	0.013	1.11 (0.73–1.68)	0.625
Marital status				
Not in a union (reference)				
In a union	0.95 (0.62–1.45)	0.798	0.44 (0.23–0.83)	0.012
Frequency of attendance at religious services				
Attends at least weekly (reference)				
Attends less often than weekly	0.70 (0.49–1.01)	0.056	0.83 (0.48–1.45)	0.512
Has had FP discussion with partner				
No (reference)				
Yes	1.92 (1.48–2.48)	0.000	4.01 (2.35–6.83)	0.000
Able to make own FP decisions				
No (reference)				
Yes	1.10 (0.79–1.53)	0.565	2.93 (1.82–4.73)	0.000
Heard FP messages in last 3 months				
No (reference)				
Yes	1.29 (0.99–1.68)	0.064	1.25 (0.81–1.92)	0.317
Desired timing of future childbearing				
More than 2 years, or does not want (reference)				
1 to 2 years	0.76 (0.57–1.00)	0.050	0.55 (0.33–0.91)	0.020
Number of FP methods received information about during non-FP visit				
None or 1 (reference)				
2+	NA		12.24 (6.48–23.10)	0.000
Number of sources FP information received from during non-FP visit				
None or 1 (reference)				
2+	NA		4.75 (2.62–8.62)	0.000
PROVIDER LEVEL VARIABLES				
Provider age				
39+ years (reference)				
38 years or less	1.00 (0.68–1.46)	0.994	0.55 (0.32–0.95)	0.032
Sex				
Male (reference)				
Female	0.73 (0.49–1.08)	0.115	0.84 (0.48–1.49)	0.555
Highest education completed				
Primary (reference)				
Secondary	2.00 (0.72–5.54)	0.184	1.23 (0.23–6.69)	0.810
College/university	3.00 (1.11–8.06)	0.030	2.20 (0.43–11.29)	0.343
Cadre				
Clinician (reference)				
Nurse/midwife	1.00 (0.62–1.63)	0.990	0.49 (0.26–0.93)	0.029
Other staff	0.87 (0.50–1.52)	0.624	0.40 (0.18–0.88)	0.023
Work hours per week				
Less than 54 h per week (reference)				
54+ hours per week	0.51 (0.35–0.74)	0.000	0.40 (0.23–0.70)	0.001
Years in cadre				
0 to 10 years				
11+ years	1.27 (0.86–1.87)	0.228	0.77 (0.44–1.36)	0.372
Years at the facility				
0 to 7 years				
8+ years	1.32 (0.88–1.99)	0.179	0.95 (0.52–1.72)	0.858
Routinely provides FP services				
No (reference)				
Yes	2.02 (1.30–3.13)	0.002	1.19 (0.61–2.33)	0.614
FP ever-training PCA score^a^				
Low (reference)				
High	1.70 (1.16–2.49)	0.007	1.53 (0.85–2.74)	0.153
FP recent-training PCA score^a^				
Low (reference)				
High	1.70 (1.14–2.53)	0.009	1.28 (0.71–2.28)	0.412
FP Practice PCA Score^a^				
Low (reference)				
High	1.64 (1.11–2.41)	0.013	1.72 (0.95–3.09)	0.071
Perception in-service training has helped job performance				
No (reference)				
Yes	2.64 (1.51–4.63)	0.001	1.75 (0.70–4.38)	0.230
Access to electronic mentoring opportunities				
No access (reference)				
Access to at least 1 type (emergency call system, e-learning, teleconference)	1.88 (1.29–0.74)	0.001	2.28 (1.31–3.98)	0.004
Job Satisfaction				
Very or a little satisfied (reference)				
Neutral or a little or very dissatisfied	0 .95 (0.65–1.38)	0.779	0.94 (0.54–1.64)	0.825
Perception of adequacy of training for job duties				
No (reference)				
Yes	1.06 (0.70–1.60)	0.780	1.31 (0.70–2.43)	0.402
Perception in-service training has helped job performance				
No (reference)				
Yes	2.64 (1.51–4.63)	0.001	1.75 (0.70–4.38)	0.230
Perception paid fairly for job duties				
No (reference)				
Yes	1.43 (0.87–2.36)	0.160	0.73 (0.33–1.58)	0.421
Displays at least one FP bias^b^				
No (reference)				
Yes	1.16 (0.78–1.71)	0.468	0.90 (0.51–1.59)	0.709

^a^Providers were asked if they had ever received training in, had recently received training in (in the last 18 months), and if they had provided the service in the last 3 months related to the following 14 FP items: 1) counsel women about FP and contraception; 2) give an injectable; 3) insert an IU(C)D; 4) remove an IU(C)D; 5) insert an implant; 6) remove an implant; 7) perform a tubal ligation; 8) perform a vasectomy; 9) provide counseling on FP options; 10) provide counseling on the efficacy of FP methods; 11) provide counseling on the potential side effects of FP methods; 12) provide counseling on the potential warning signs of FP methods; 13) provide clinical management of FP side effects; and 14) provide FP for HIV positive women

^b^Providers were asked if they decide whether or not to provide FP services to clients based on one or more of the following client characteristics: marital status/parental consent/partner consent/age/parity

## Abbreviations

95% CI: 95% Confidence intervals
ANC: Antenatal care
BEmONC: Basic Emergency Obstetric and Neonatal Care
BRN: Big Results Now
CDC: Centers for Disease Control and Prevention
CEmONC: Comprehensive Emergency Obstetric and Neonatal Care
DRH: Division of Reproductive Health
FP: Family planning
ICC: Intraclass correlation
IUD: Intrauterine Device
LAM: Lactational amenorrhea method
MOHCDGEC: Ministry of Health, Community Development, Gender, the Elderly, and Children
NBS: National Bureau of Statistics
OR: Odds Ratios
PCA: Principal Components Analysis
PL: Pregnancy loss
PNC: Postnatal Care
TFR: Total Fertility Rate
WHO: World Health Organization

## Footnotes

1 Providers were asked if they decide whether or not to provide FP services to clients based on one or more of the following client characteristics: marital status/parental consent/partner consent/age/parity

2 Providers were asked if they had ever received training in, had recently received training in (in the last 18 months), and if they had provided the service in the last 3 months related to the following 14 FP items: 1) counsel women about FP and contraception; 2) give an injectable; 3) insert an IU(C)D; 4) remove an IU(C)D; 5) insert an implant; 6) remove an implant; 7) perform a tubal ligation; 8) perform a vasectomy; 9) provide counseling on FP options; 10) provide counseling on the efficacy of FP methods; 11) provide counseling on the potential side effects of FP methods; 12) provide counseling on the potential warning signs of FP methods; 13) provide clinical management of FP side effects; and 14) provide FP for HIV positive women.
